# The efficacy of virtual reality technology for the postoperative rehabilitation of patients with cervical spondylotic myelopathy (CSM): a study protocol for a randomized controlled trial

**DOI:** 10.1186/s13063-024-07962-9

**Published:** 2024-02-19

**Authors:** Jiajun Wu, Zhongchuan Sun, Zhichao Ge, Dong Zhang, Jianghan Xu, Rilin Zhang, Xuecheng Liu, Qing Zhao, Hao Sun

**Affiliations:** 1 Department of Spinal Surgery, Yueyang Hospital of Integrated Traditional Chinese and Western Medicine, Shanghai University of Traditional Chinese Medicine, Shanghai, 200437 China; 2 Center of Rehabilitation Medicine, Yueyang Hospital of Integrated Traditional Chinese and Western Medicine, Shanghai University of Traditional Chinese Medicine, Shanghai, 200437 China

**Keywords:** Virtual reality technology, Cervical spondylotic myelopathy, Postoperative rehabilitation, Brain plasticity, Randomized controlled trial

## Abstract

**Background:**

Patients with cervical spondylosis myelopathy (CSM) may experience severe neurological dysfunction due to untimely spinal cord compression after surgery. These disorders may lead to sensory and motion disorders, causing considerable psychological distress. Recent studies found that virtual reality (VR) technology can be an effective tool for treating spinal cord injuries. Owing to this discovery, we developed an exploratory research project to investigate the impact of this intervention on the postoperative recovery of patients with CSM.

**Methods:**

The purpose of this randomized controlled trial was to evaluate the efficacy of combining VR technology with conventional rehabilitation strategies for the postoperative rehabilitation of patients with CSM. A total of 78 patients will be recruited and randomized to either the conventional rehabilitation group or the group subjected to VR technology combined with conventional rehabilitation strategies. The Japanese Orthopaedic Association (JOA) scale will be the main tool used, and secondary outcomes will be measured via the visual analogue scale (VAS), neck disability index (NDI), and functional MRI (fMRI). The data analysis will identify differences between the intervention and control groups as well as any relationship between the intragroup changes in the functional area of the brain and the subjective scale scores after the intervention.

**Discussion:**

The aim of this trial is to investigate the effect of VR training on the postoperative rehabilitation of patients with CSM after 12 intervention treatments. Positive and negative outcomes will help us better understand the effectiveness of the intervention and its neural impact. If effective, this study could provide new options for the postoperative rehabilitation of patients with CSM.

**Trial registration:**

Chinese Clinical Trial Registry (ChiCTR2300071544). Registered 17 May 2023, https://www.chictr.org.cn/.

**Supplementary Information:**

The online version contains supplementary material available at 10.1186/s13063-024-07962-9.

## Background

Cervical spondylotic myelopathy (CSM) is a spinal cord disease characterized by degenerative changes in the cervical spine, such as degenerative disc disease, ossification of the posterior longitudinal ligament (OPLL), and hypertrophy of the yellow ligament. These changes can cause the surrounding blood vessels and nerve structures to become compressed, thus leading to a range of sensory and motor symptoms. The clinical symptoms included limb weakness, gait instability, and diminished sensory and motor function. In severe cases, quadriplegia can occur. Moreover, CSM is more common in middle-aged and elderly individuals older than 55 years [1]. In North America, the incidence and prevalence of CSM are at least 41 and 605 per million people, respectively, while in East Asian countries, the hospitalization rate for patients with CSM is 4.04 per 100,000 person-years [2, 3]. With social progress, technological advancements, and changes in human lifestyles and work patterns, the incidence of CSM is increasing annually, particularly in young people [4]. Surgery is the most effective treatment for patients with middle and advanced stage disease [5]. However, some postoperative CSM patients still experience sensory and motor disorders, such as lower limb weakness, gait instability, and numbness, which need to be addressed. With the development of neuroimaging, researchers studying brain plasticity found that CSM could lead to brain plasticity, which plays an important role in functional recovery after surgery for CSM [6]. A deeper understanding of the development of CSM-related brain plasticity is highly important for developing appropriate rehabilitation plans and improving clinical efficacy.

The central nervous system (CNS) has been confirmed to be able to reorganize structurally and functionally after injury, enabling repair through neuroplasticity [7]. Brain plasticity is the brain’s ability to modify its structure and function to adapt to environmental changes and experiences [8]. Researchers revealed that the recovery of neural function in CSM patients depends on both spinal cord compression and injury as well as brain function reorganization or plasticity [9]. Moreover, cortical plasticity compensates for some function by involving brain regions that are not primarily involved in a specific task [10]. Therefore, when spinal cord injury occurs, cortical plasticity also occurs. Sabbah, P. et al. discovered that cortical reorganization, either physically or via mental imagery, could activate the lower limb cortical network years after injury and is therefore an adaptive remodelling response known as cortical-spinal neural plasticity [11]. Liu, M. et al. conducted a study using volumetric magnetic resonance imaging (MRI) with voxel-based morphometry (VBM) on 41 CSM patients both before and after surgery and compared the VBM measurements to those of a control group [12]. They assessed the changes in the cortical grey matter volumes in CSM patients before and after surgery and found that, compared to those in the control group, the grey matter volumes in the left caudate nucleus and the right thalamus were lower in the preoperative CSM patients. Six months after surgery, the bilateral grey matter volume in the posterior cerebellar lobes decreased in the postoperative CSM patients, while the grey matter volume in the brainstem was greater than that in the preoperative CSM patients. Compared to the control group, the postoperative CSM patients in the present study had a significantly lower grey matter volume in the left caudate nucleus but higher grey matter volumes in the right inferior temporal gyrus, right orbitofrontal cortex (OFC), and bilateral lingual gyri/cuneus/precuneus cortex. Therefore, based on the scientific theory of brain plasticity, it is important to select appropriate methods to regulate the CNS and promote spinal cord recovery after surgery to ensure the smooth rehabilitation of CSM patients. In recent years, VR technology has been increasingly applied in clinical treatment, particularly for incomplete spinal cord injury, stroke, Parkinson’s disease, and other areas, to promote rehabilitation through brain remodelling [13–15]. This technology could also be a potential therapeutic option for CSM patients after surgery.

VR technology is a tool that allows users to create and experience computer-generated 3D environments and interact with various objects in real time through visual, auditory, or tactile feedback. VR is a simulated 3D interactive system with dynamic scenes and entity behaviours that allows the integration of multiple sources of information such as scene displays, force/tactile sensors, and position trackers. Motion training has shown promise in that patients can become immersed in virtual environments, thereby providing a pleasant and exciting experience for users. Multisensory stimulation provided by VR can potentially promote the reshaping and reconstruction of the central nervous system, improving gait instability. Kirsch, P et al. used fMRI technology to demonstrate that monetary rewards produced greater brain activation than verbal rewards, suggesting that the type of reward has a strong stimulating effect on brain activity [16]. Animal and human neuroimaging studies have shown that reward delivery is associated with the activation of the subcortical edge and anterior brain areas, including the substantia nigra, thalamus, striatum, orbital frontal cortex, insula, and precentral gyrus. The activation of these relevant brain regions through the reward system may lead to brain reshaping. The degree of motor function recovery depends on the extent of neural population reshaping induced by the intervention. Neuroimaging studies have demonstrated that VR can induce cortical reshaping of motor pathways, including the primary sensory motor cortex, SMA, cerebellum, precentral gyrus, and ipsilateral and contralateral marginal gyri, through visual, auditory, and tactile feedback [17]. This approach creates a realistic environment and provides a safe and stimulating learning environment for subjects. By stimulating brain plasticity through a large number of movement repetitions and multisensory methods, brain reshaping mechanisms can be used to reconstruct gait.

### Study aim

The main objective of this randomized controlled trial was to evaluate the effectiveness of virtual reality technology in the postoperative rehabilitation of patients with CSM. The secondary objective was to explore the potential patterns of cortical plasticity related to sensory and motor function recovery in CSM patients via pre- and postintervention fMRI and longitudinal JOA studies to provide a theoretical basis for investigating the central role of virtual reality training in promoting brain plasticity in patients who have undergone surgery for cervical spondylosis.

## Method/design

### Study design

**Fig. 1:**
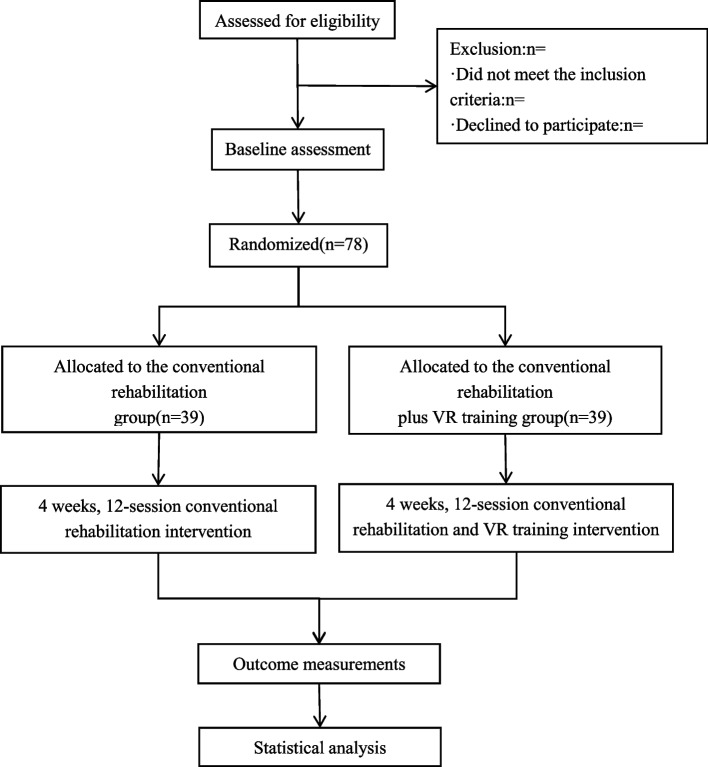
Flow chart of study design

**Fig. 2:**
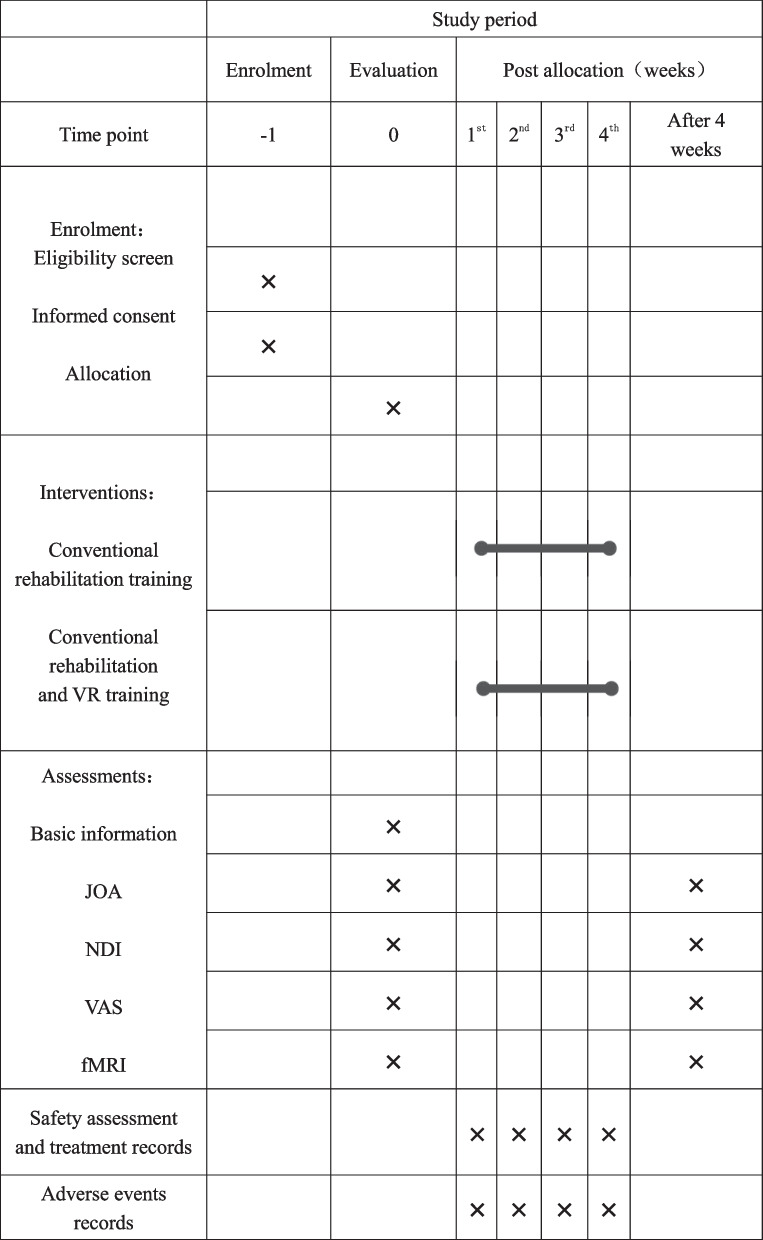
The schedule of enrolment, interventions, and assessments. Abbreviations: JOA, Japanese Orthopaedic Association; NDI, Neck Disability Index; VAS, visual analogue scale; fMRI, functional magnetic resonance imaging

The aim of this randomized controlled trial was to investigate the effectiveness of virtual reality (VR) technology in the postoperative rehabilitation of patients with CSM. A total of 78 eligible patients were recruited and randomly assigned to either the routine rehabilitation group or the VR training combined with routine rehabilitation group (1:1). The primary outcome will be measured with the Japanese Orthopaedic Association (JOA) (Table 1) cervical myelopathy evaluation scale, and the secondary measures will include the visual analogue scale (VAS), neck disability index (NDI), and functional magnetic resonance imaging (fMRI) scans [18–20]. The intervention will be implemented in accordance with a standardized protocol, and independent researchers will execute and supervise the random allocation of patients in the trial. The study protocol is in compliance with the SPIRIT recommendation. For the SPIRIT checklist, see Additional file 1, and the study design is presented in Figs. 1 and 2. This study is an exploratory investigation.

**Table 1:** Japanese Orthopaedic Association (JOA) score for patients with cervical myelopathic

Motor function	
Upper extremity	
The thumb and fingers	
0 [Complete disturbance] The patient cannot use chopsticks or a spoon/fork, and cannot fasten a button on his or her own.	
1 [Severe disturbance] The patient cannot use chopsticks or write, and can barely use a spoon/fork.	
2 [Moderate disturbance] The patient can pick up a large object with chopsticks but can hardly write. He/she can fasten a large button.	
3 [Slight disturbance] The patient makes awkward use of chopsticks, writes in a clumsy manner, but can fasten buttons on his/her shirt.	
4 [Normal] Normal	
Lower extremity	
0 [complete disturbance] The patient cannot stand or walk alone.	
(0.5 The patient can stand up.)	
1 [Severe disturbance] The patient needs support to walk on a flat surface.	
(1.5 The patient can walk on a flat surface without any support but the walking is not stable.)	
2 [Moderate disturbance] The patient can walk on a flat surface without any support, but needs a handrail to walk up and down stairs.	
(2.5 The patient can walk on a flat surface without any support, but needs a handrail only to walk down stairs.)	
3 [Slight disturbance] The patient can walk fast, although awkwardly.	
4 [Normal] Normal	
Sensory function	
Upper extremity	
0 [Severe disturbance] Complete sensory loss (touch sensation, pain sensation)	
(0.5 Partial sensory loss ≤ 5/10 (touch sensation, pain sensation); intolerable pain or numbness)	
1 [Moderate disturbance] Partial sensory loss ≥ 6/10 (touch sensation, pain sensation); numbness and hypersensitivity.	
(1.5 [Slight disturbance] Slight numbness (normal sensation))	
2 [Normal] Normal	
Trunk	
0 [Severe disturbance] Complete sensory loss (touch sensation, pain sensation)	
(0.5 Partial sensory loss ≤ 5/10 (touch sensation, pain sensation); intolerable pain and numbness)	
1 [Moderate disturbance] Partial sensory loss ≥ 6/10 (touch sensation, pain sensation); numbness and hypersensitivity.	
(1.5 [Slight disturbance] Slight numbness (normal sensation))	
2 [Normal] Normal	
Lower extremity	
0 [Severe disturbance] Complete sensory loss (touch sensation, pain sensation)	
(0.5 Partial sensory loss ≤ 5/10 (touch sensation, pain sensation); intolerable pain and numbness)	
1 [Moderate disturbance] Partial sensory loss ≥ 6/10 (touch sensation, pain sensation); numbness and hypersensitivity.	
(1.5 [Slight disturbance] Slight numbness (norm sensation))	
2 [Normal] Normal	
Urinary bladder function	
0 [Severe disturbance] Urinary retention, incontinence	
1 [Moderate disturbance] Feeling of residual urine, straining of oneself, dull urination, elongation of urination (retarded urination), urinary incontinence	
2 [Slight disturbance] Retarded urination, pollakisuria	
3 [Normal] normal	
Total 17

### Participants

The aim of this study was to recruit 78 patients who underwent surgery for CSM, with JOA scores between 5 and 15 and aged between 20 and 75 years. All eligible participants will be recruited from the orthopaedic and rehabilitation medicine departments of our hospital and other hospitals. The study was conducted at multiple centres, including Shanghai Longhua Hospital, Affiliated with Shanghai University of Traditional Chinese Medicine, and Shanghai Shuguang Hospital, Affiliated with Shanghai University of Traditional Chinese Medicine. Both of these hospitals are located in Shanghai, China. Prior to the experiment, we provided a comprehensive explanation of the study’s purpose, type of intervention, and potential risks and benefits of using VR technology to the participants who consented to participate. We will respect their decision to withdraw from the study at any time for any reason. At the same time, we maintained regular contact with participants, created incentives, provided personalised designs, simplified experiment procedures, and increased communication and engagement to increase loyalty and participation and to ensure that the experiment would be successful.

## Enrolment

### Inclusion criteria

All participants who met the following criteria were included: (1) diagnosed with CSM and underwent surgery between 24 h and 3 months prior to enrolment; (2) aged between 20 and 75 years of either sex; (3) a Japanese Orthopaedic Association (JOA) score of 5 to 15; (4) no less than a primary school education; (5) no cognitive dysfunction; and (6) understood the study, agreed to participate in the study, and signed the informed consent form.

### Exclusion criteria

Participants were excluded if they met any of the following conditions: (1) contraindications for magnetic resonance imaging (such as a cardiac pacemaker, cardiac stent, artificial heart valve, bone fracture fixation plate, or claustrophobia); (2) concurrent limb fractures, degenerative diseases of the lumbar or thoracic vertebrae, cerebrovascular disease, other neurological disorders, intraspinal space-occupying lesions, history of cervical trauma, previous cervical spine surgery, hip or knee joint diseases, or other serious systemic diseases; (3) severe cardiopulmonary dysfunction; or (4) pregnant or lactating.

### Withdrawal or dropout criteria

In this study, the intervention measures for both the trial group and the control group may be stopped if the subject meets any of the following criteria: (1) the subject requests to withdraw from the clinical study; (2) the subject is unable to complete the relevant test as needed, which affects the data collection; (3) the subject receives other treatments during the treatment period without authorization; (4) the subject experiences adverse events or serious adverse events during the trial that require their withdrawal from the study; (5) the subject experiences significant worsening of the disease, complications or special physiological changes that require their withdrawal from the study; (6) the researcher considers it unsuitable for the subject to continue participating in this study.

### Who will provide informed consent?

All eligible postoperative patients with cervical spondylotic myelopathy (CSM) were informed about the methods and objectives of this study as well as the potential benefits and risks involved. Patients were provided with an informed consent form for review and signing.

### Intervention

Interventions using VR technology were conducted on the GRAIL system (Netherlands Motek Medical Company SY02097646), with all participants undergoing diagnostic assessment by a professional spine specialist or rehabilitation medicine physician prior to participation to ensure suitability. The training time was scheduled in accordance with the GRAIL guidelines to ensure that the VR intervention was appropriate and safe, as it has been shown to be the most effective intensity of training for patients with CSM. All intervention measures were implemented by qualified therapists, and the participants’ reactions were recorded after each intervention. Safety measures were taken to prevent harm to all participants.

### Conventional rehabilitation group

Patients in the rehabilitation group underwent a programme that focused primarily on strength training to improve their performance ability and coordination [21]. The postoperative limb muscle strength, sensation, and motor function of patients was assessed to develop a functional training program based on existing problems. The program included the following exercises: (1) finger extension and forceful bending and gripping movements were repeated 5–10 times, and the frequency of training sessions was appropriately increased. (2) Patients were guided in performing active knee and hip joint movements 3 times a week for 15 min each 24 h after surgery. (3) Straight leg lift exercises were performed 2 times a week for 15 min each. (4) Isometric contraction exercises, such as those involving the quadriceps femoris, were performed 3 times a week for 10 min each. (5) Ankle joint dorsiflexion and plantar flexion exercises were performed 3 times a week for 15–20 min each. (6) Walking training was performed 3 times a week for 10–20 min each time.

### Conventional rehabilitation and virtual reality training group

The same specific plan as previously described was used for conventional rehabilitation treatment. Additionally, virtual reality technology was used to supplement the GRAIL procedure. The treatment plan involved instructing patients to perform corresponding activities on a treadmill while being suspended by a harness and surrounded by suitable virtual scenes. The frequency of treatment was three times a week, with each session lasting 15–20 min. This treatment lasted for 4 weeks, during which time a doctor was present to guide and monitor the patient throughout the sessions.

The virtual reality rehabilitation sessions will be conducted using a commercially available VR system. Participants in the intervention group will wear a VR headset and engage in various virtual reality activities that target specific motor and functional aspects affected by CSM. These activities will include exercises and games designed to improve range of motion, strength, coordination, and balance.

### Outcomes

Prior to the intervention, individuals who expressed interest in participating in the study were invited to schedule an appointment for additional information.

If they choose to participate, they will sign an informed consent form and be screened by independent researchers to determine if they meet the inclusion and exclusion criteria. Participants who meet all the inclusion criteria and none of the exclusion criteria will be included in the study. Descriptive data, including general information such as age, sex, race, occupation, height, weight, body mass index (BMI), blood pressure, education level, medical history, medication use, smoking status, drug use, and alcohol consumption, will be carefully collected before randomization. In addition, all participants were assessed using three scales: the JOA, the VAS, and the NDI.

Both the intervention group and the control group completed a total of 12 rehabilitation sessions over a period of 4 weeks. Outcomes will be assessed immediately after the 4-week intervention period and compared with baseline data. The primary outcome will be the change in functional ability, assessed using a standardized functional assessment tool (JOA score). The secondary outcome measures will include pain level (VAS score), neck disability (NDI score), and changes in the functional areas of the brain.

### Measurements

Figure 2 lists the schedule of all study assessments and the timeframe in detail. The assessments are described briefly below:

#### Primary outcome measurement

The primary outcome measure was the change in the JOA score for the cervical spine. The JOA score consists of a total of 17 points, with 4 points each for upper and lower limb motor function, 2 points each for upper and lower limb and trunk sensation, and 3 points each for bladder function. Patients were assessed using the JOA score before intervention, after intervention, and during follow-up in accordance with the JOA evaluation criteria for cervical spine disease treatment established by the Japanese Orthopaedic Association. The results were categorized into five groups: > 75% excellent, 50–74% good, 20–49% fair, 0–19% no change, and < 0 deterioration.

#### Secondary outcome measures

The secondary outcome measures consisted of the VAS score, NDI score, and neuroimaging data. The data will be collected at the baseline assessment and at the end of the intervention (4 weeks after randomization).

#### Visual analogue scale (VAS)

The VAS pain consists of a 100-mm horizontal line with the words “no pain” and “worst possible pain” at the line’s ends. Patients were asked to quantify their neck pain by drawing a vertical mark on the area of the horizontal line that best represented their pain level during the preceding 24 h.

#### Neck disability index (NDI) score

The NDI measures disability related to neck pain and includes 10 items related to neck pain intensity, personal care, lifting, reading, headache, concentration, working, sleeping, driving, and recreation. Each item is scored from 0 to 5, with higher scores indicating more severe disability. The results are interpreted as follows: 0–20% indicates mild disability, 21–40% indicates moderate disability, 41–60% indicates severe disability, 61–80% indicates very severe disability, and 81–100% indicates complete disability or the need for further examination to rule out symptom exaggeration.

#### Neuroimaging scans

The data for this study were collected at the Radiology Department of Yueyang Hospital of Integrated Traditional Chinese and Western Medicine, which is affiliated with the Shanghai University of Traditional Chinese Medicine. Participants were positioned in the supine position, and their heads were secured using a specially designed head mask. All magnetic resonance imaging (MRI) scans were performed using a Siemens Magnetom Verio 3. T MRI scanner. Resting-state functional MRI (fMRI) data were obtained using a single-shot echo-planar imaging (EPI) sequence with interleaved scanning of 43 slices, a flip angle of 90°, a matrix of 64 × 64, a TR of 300 ms, a slice thickness of 3.0 mm, an FOV of 230 × 230 mm^2^, and a gap of (voxel size of 3.6 × 3.6 × 3.0 mm^3^). T1-weighted structural scans were obtained using a weighted fast spoiled gradient-echo (FSPGR) sequence with TR/TI/TE of 190/900/2.93 ms, a flip angle of 9°, an FOV of 256 × 256 mm^2^, a slice thickness of 1.0 mm, a matrix of 256 × 256, and an average of 1.0. All assessments involving scales and MRI scans were conducted prior to the intervention and then repeated after 4 weeks of intervention.

### Safety assessment

Any adverse events such as discomfort caused by MRI procedures, headache, or VR training that cannot be tolerated will be recorded throughout the intervention period and reported to the ethics committee to ensure safety. Operators conducting GRAIL procedures should be familiar with the trial protocol and procedures related to safety, and ethics must strictly follow the Grail operation guidelines. In case of an adverse event during the intervention, the participant will receive appropriate medical care, and all the details will be recorded and reported to the principal investigator and ethics committee to determine if the participate should continue the trial. All collected adverse events will be truthfully reported in future publications, and the incidence rate of adverse events will also be considered in the analysis.

### Sample size calculation

The goal of our study was to investigate the effectiveness of VR technology in the rehabilitation of patients with CSM after surgery. Due to a lack of research on the effect of VR technology on the rehabilitation of CSM patients after surgery, we conducted a small-sample pilot study. The sample size was estimated based on the improvement in JOA scores for cervical spondylosis reported by the Japanese Orthopaedic Association. One month after surgery, the average JOA scores and SDs for the VR rehabilitation intervention group (*n* = 10) and the conventional rehabilitation group (*n* = 10) were 15.30 (SD = 0.90) and 14.7 (SD = 0.78), respectively, with an effect size (Cohen’s *D*) of 0.71. Using the Gpower 3.1 software, we calculated the needed sample size, which, with a type I error of *α* = 0.05 and a type II error of *β* = 0.2, was estimated to be a sample size of *n* = 64. Considering a dropout rate of 20%, 39 participants were needed for each group, for a total of 78 participants. Finally, we randomly allocated the 78 participants into a VR training combined with rehabilitation intervention group and a conventional rehabilitation group, each consisting of 39 participants.

### Randomization and allocation concealment

After the baseline evaluation, each eligible participant was randomly assigned to either the intervention group or the control group at a 1:1 ratio. The random allocation cards will be generated using the random permutation principle with a random number table generated by SPSS21. These cards will be placed in opaque envelopes, with the envelope numbers corresponding to the card numbers. The envelopes will be opened in the order of the participants’ visits, and the participants will be randomly assigned to either the conventional rehabilitation group or the conventional rehabilitation combined with VR training group based on the grouping specified on the card inside the envelope. This method of random allocation will ensure that the assignment is unbiased and that each participant has an equal chance of being assigned to either group.

## Blinding

### Who will be blinded

For this trial, the individuals responsible for brain functional magnetic resonance imaging (fMRI) data collection and analysis were kept blinded. A physiotherapist who was involved in the treatment of spinal disorders and belonged to the same institution as the first author was the therapist. Patients, therapists, and evaluators were not blinded to the treatment group to which the patients had been allocated.

### Procedure for unblinding

The unblinding procedure was conducted at the completion of the trial or at the specified endpoint. An independent unblinding committee composed of experts unrelated to the study was established. The committee members were blinded to the allocation of participants throughout the trial to maintain independence and objectivity. A detailed unblinding plan, including the timing, sequence, and identity verification process of unblinded participants, was developed and approved by the ethics committee of Yueyang Integrated Chinese and Western Medicine Hospital affiliated with Shanghai University of Traditional Chinese Medicine prior to trial initiation. Randomization codes and group assignments were securely stored by an independent data manager or committee member. The unblinding process was executed by members of the unblinding committee or by authorized individuals; this process involved the verification of participants’ group assignments and the provision of treatment codes or the decryption of treatment groups. After unblinding, the data analysts analysed the data and interpreted the trial results based on the unblinded data, including comparisons and assessments of statistical significance between the treatment and control groups.

### Data collection and management

Initially, the screening personnel reviewed the inclusion and exclusion criteria and collected basic characteristic data prior to random allocation. Trained assessors, who are blinded to the allocation and not involved in the intervention, will play a crucial role in measuring the primary and secondary outcomes. The data for all participants will be recorded in a designed case report form (CRF). Additionally, each CRF will be checked twice to ensure the accuracy and completeness of the data collection throughout the study. All relevant documents will be coded with specific identification numbers and kept in a secure, locked box to safeguard the privacy and data safety of the subjects. The magnetic resonance scans obtained during the study will be saved on a computer with restricted access and a password known only to the principal investigator. The materials will be securely stored in compliance with medical records retention regulations and good clinical practice principles for 5 years after the trial. The study will also be monitored for safety by a physical therapist and two clinical doctors who are not involved in the research. The researchers will ensure that the study is conducted in compliance with standard operating procedures (SOPs), monitor the study’s progress, review all adverse events, and discontinue the trial if any measures cause significant adverse effects such as headache, nausea, or vomiting. The assessors reported any adverse events to the *data monitoring committee* (DMC) and the ethics committee of Yueyang Integrated Chinese and Western Medicine Hospital affiliated with Shanghai University of Traditional Chinese Medicine. DMC is independent from the sponsor and competing interests.

### Statistical analysis

All nonimaging data will be analysed using SPSS version 21 by an independent statistician who is blinded to the treatment and control groups to ensure objectivity and reduce bias. The statistical significance level for testing will be set at 0.05 with a two-tailed test. Continuous variables are presented as the mean, standard deviation, or maximum or minimum values based on their statistical distributions. Independent sample *t* tests or nonparametric tests (Mann–Whitney *U* test) will be used to compare the results presented as continuous variables and mean values to validate the effectiveness of the intervention. Furthermore, intra- and intergroup comparisons were made at 4 weeks to observe the differences between the two intervention methods. If necessary, logistic regression, general linear correlation, or regression was used to compare different types of results based on the variable type and distribution. Basic data comparisons between the treatment and control groups will be performed using *t* tests, chi-square tests, or nonparametric tests. Participants who drop out during the intervention period will not be excluded according to the intention-to-treat principle, and missing data will be replaced using the last observation carried forward method to maintain statistical power and avoid bias.

The amplitude of low-frequency fluctuation (ALFF) is an indicator of the amplitude energy of the low-frequency spectrum and reflects the spontaneous activity of neurons in the resting state of the brain. The ALFF is a useful tool for analysing functional MRI data to study longitudinal neural changes and explore the relationship between brain plasticity and the recovery of sensory and motor function induced by stimulation [22]. In this study, the REST 1.9 software from Beijing Normal University (http://www.restfmri.net/) was used to perform linear drift and calculate the amplitude of signal oscillation in each voxel in the low-frequency range (0.01–0.1 Hz) to obtain ALFF images. Finally, the ALFF values of each participant were *Z*-transformed for further statistical analysis.

Longitudinal measurements in a resting-state task and further correlation analysis of imaging and scale score data obtained from baseline and postintervention measurements were performed to explore the relationships between brain remodelling and postoperative sensory and motor function recovery in CSM patients.

### Protocol amendments

This trial will be conducted in accordance with Protocol Version 1.0. Any modifications to the protocol will be formally amended and submitted to the Ethics Committee of Yueyang Hospital of Integrated Traditional Chinese and Western Medicine, affiliated with Shanghai University of Traditional Chinese Medicine. The study will be conducted in compliance with the principles of the Declaration of Helsinki and Good Clinical Practice guidelines. Any deviations from the protocol will be documented and reported to the Ethics Committee.

### Dissemination plans

The results of this study will be accessible in peer-reviewed publications and presented at academic conferences.

## Discussion

CSM is the most severe type of cervical spondylosis, and in severe cases, it can lead to paralysis. Surgery is the only reliable solution for patients with advanced CSM who suffer from severe sensory and motor dysfunction, such as lower limb weakness, limb numbness, unstable gait, and limited fine motor activity of the hands. However, even after relieving the pressure on the spinal cord caused by prolonged compression, damaged nerve function may not fully and quickly recover. As a result, residual sensory and motor impairments after surgery can cause significant psychological stress. Rehabilitation is considered an important component of recovery in many neurological disorders, including stroke, traumatic brain injury (TBI), and spinal cord injury (SCI) [15, 23, 24]. Despite these findings, there has been a lack of clinical research promoting rehabilitation for CSM patients [25]. New evidence suggests that rehabilitation strategies may have a substantial impact on the recovery of CSM patients, and existing rehabilitative interventions for traumatic SCI and stroke may be applicable due to their similar pathophysiology [26]. Various rehabilitation methods can be used for the postoperative rehabilitation of patients with CSM, such as gait training, hand function training, and VR training. The latter is particularly interesting due to its multisensory stimulation, which promotes brain reshaping and functional recovery, integrating traditional gait training into more enjoyable VR training. Additionally, numerous technologies, such as transcranial magnetic stimulation (TMS) and transcranial direct current stimulation, have been rapidly developed as noninvasive neuroregulation techniques. These techniques have been widely used in the clinical treatment of various neurological disorders, such as stroke, spinal cord injury, Alzheimer’s disease, Parkinson’s disease, chronic pain, depression, and addiction, with promising results [27–29]. No relevant literature on postoperative neuroregulation treatments for CSM patients at the brain functional level have been found. However, we believe that using noninvasive neuroregulation techniques to purposefully regulate brain function after effective cervical spine surgery decompression could be highly important for improving its short-term efficacy and the long-term recovery of CSM patients. One potential limitation of this trial is that it was not a double-blind controlled trial. Although we attempted to blind everyone, this approach is not always feasible in nonpharmacological studies, and we cannot expect participants in the trial to pretend to be blind to the recovery treatment or to the therapists and clinicians [30]. However, throughout the trial, outcome assessors or statisticians will be blinded to the study design to eliminate any potential bias.

Overall, the aim of this randomized clinical trial is to investigate the potential of 12 sessions of VR stimulation in promoting gait stability while also seeking to elucidate the underlying mechanisms of this stimulation. This research will fill the research gap regarding the postoperative rehabilitation of patients with cervical spondylosis using VR training. These positive results from our trial may provide new treatment options and evidence for improving gait instability.

## Trial status

This is version 2.0 of the protocol, dated 30 May 2023. Participant recruitment began on 7 June 2023. Recruitment is predicted to continue until December 2024. Currently, recruitment is still ongoing at the time of submission.

### Supplementary Information

**Additional file 1. **SPIRIT Checklist for *Trials.*

## Data Availability

Upon completion of the trial, only researchers or teams with ethical approval will have access to the final datasets. The datasets analysed during the current study will be available from the corresponding author upon reasonable request.

## Abbreviations

CSM: Cervical spondylosis myelopathic
VR: Virtual reality
JOA: Japanese orthopaedic association
VAS: Visual analogue scale
NDI: Neck disability index
fMRI: Functional magnetic resonance imaging
OPLL: Ossification of the posterior longitudinal ligament
CNS: Central nervous system
MRI: Magnetic resonance imaging
VBM: Voxel-based morphometry
OFC: Orbitofrontal cortex
CRF: Case report form
SOPs: Standard operating procedures
DSMB: Data safety monitoring board
ALFF: Amplitude of low frequency fluctuation
BMI: Body mass index
TBI: Traumatic brain injury
SCI: Spinal cord injury
TMS: Transcranial magnetic stimulation
